# Triple negativity and young age as prognostic factors in lymph node-negative invasive ductal carcinoma of 1 cm or less

**DOI:** 10.1186/1471-2407-10-557

**Published:** 2010-10-15

**Authors:** Ji Hyun Kwon, Yu Jung Kim, Keun-Wook Lee, Do-Youn Oh, So Yeon Park, Jee Hyun Kim, Eui Kyu Chie, Sung-Won Kim, Seock-Ah Im, In-Ah Kim, Tae-You Kim, In Ae Park, Dong-Young Noh, Yung-Jue Bang, Sung Whan Ha

**Affiliations:** 1 Department of Internal Medicine, Seoul National University College of Medicine, Seoul National University Bundang Hospital, Seongnam, Korea; 2 Department of Internal Medicine, Seoul National University College of Medicine, Seoul National University Hospital, Seoul, Korea; 3 Department of Pathology, Seoul National University College of Medicine, Seoul National University Bundang Hospital, Seongnam, Korea; 4 Department of Radiation Oncology, Seoul National University College of Medicine, Seoul National University Hospital, Seoul, Korea; 5 Department of Surgery, Seoul National University College of Medicine, Seoul National University Bundang Hospital, Seongnam, Korea; 6 Department of Radiation Oncology, Seoul National University College of Medicine, Seoul National University Bundang Hospital, Seongnam, Korea; 7 Department of Pathology, Seoul National University College of Medicine, Seoul National University Hospital, Seoul, Korea; 8 Department of Surgery, Seoul National University College of Medicine, Seoul National University Hospital, Seoul, Korea

## Abstract

**Background:**

Whether a systemic adjuvant treatment is needed is an area of controversy in patients with node-negative early breast cancer with tumor size of ≤1 cm, including T1mic.

**Methods:**

We performed a retrospective analysis of clinical and pathology data of all consecutive patients with node-negative T1mic, T1a, and T1b invasive ductal carcinoma who received surgery between Jan 2000 and Dec 2006. The recurrence free survival (RFS) and risk factors for recurrence were identified.

**Results:**

Out of 3889 patients diagnosed with breast cancer, 375 patients were enrolled (T1mic:120, T1a:93, T1b:162). Median age at diagnosis was 49. After a median follow up of 60.8 months, 12 patients developed recurrences (T1mic:4 (3.3%), T1a:2 (2.2%), T1b:6 (3.7%)), with a five-year cumulative RFS rate of 97.2%. Distant recurrence was identified in three patients. Age younger than 35 years (HR 4.91; 95% CI 1.014-23.763, p = 0.048) and triple negative disease (HR 4.93; 95% CI 1.312-18.519, p = 0.018) were significantly associated with a higher rate of recurrence. HER2 overexpression, Ki-67, and p53 status did not affect RFS.

**Conclusions:**

Prognosis of node-negative breast cancer with T1mic, T1a and T1b is excellent, but patients under 35 years of age or with triple negative disease have a relatively high risk of recurrence.

## Background

Owing to the increased use of screening mammography, the proportion of early stage breast cancer is increasing internationally [1-3]. In Korea, an increase in the percentage of stage 0 and I types of breast cancer has also been reported. The proportion of stage 0 breast cancer was 7.5% in 2002 and increased to 11.3% in 2003. For stage I breast cancer, the proportion of patients increased from 29.5% to 36.5% over the same period [4,5].

Generally, it has been reported that the prognosis of small-sized, node-negative breast cancer is excellent with complete surgical resection of the primary tumor, even without systemic adjuvant therapy [6]. For patients with microinvasive breast cancer, which is defined as tumor foci of 0.1 cm or less, a very small percentage of women relapse or die of breast cancer [7]. According to an analysis of the Surveillance Epidemiology and End Results (SEER) data from 1998 to 2001, the ten-year overall breast cancer specific mortality of patients with T1a, bN0M0 disease was 4% [8].

Nevertheless, certain subgroups of patients who were initially diagnosed with small, node-negative breast cancer tumors have a risk of recurrence. Some authors have reported that young patients under 35 years old had poor prognosis [9]. The biological factors of a high grade of tumor [8,10,11] and high Ki-67 [12] were associated with a high relapse or high mortality rate.

There have been few clinical studies with large numbers of patients over a long duration of observation sufficient for the evaluation of the prognosis of node-negative breast cancer of 1cm or less in size. In addition, few studies have evaluated the long-term prognosis according to the hormone receptor and human epidermal growth factor receptor 2 (HER2) statuses because many patients were not examined for estrogen receptor (ER), progesterone receptor (PR), or HER2 status before these tests became widely available. Therefore, some controversy remains regarding treatment decisions for these patients.

The features of breast cancer vary among Asian countries, but there is a common tendency of younger age-onset and a large proportion of premenopausal women [5,13]. Some studies have reported higher rates of hormone receptor negative or high-grade breast tumor in Asian populations [14]. However, the ethnic differences associated with recurrence or mortality remain unclear, especially in node-negative early breast cancer of 1cm or less.

The aim of this study was to evaluate the overall prognosis of lymph node negative invasive ductal carcinoma of the breast of ≤ 1 cm in size, including microinvasive carcinoma (T1mic) in a Korean population. Moreover, through identification of subgroups of patients with a high risk of recurrence and their characteristics, we set out to determine which subgroup of patients would be candidates for systemic adjuvant therapies.

## Methods

### Study population

We identified the study population from a prospectively maintained hospital-based cancer registry and collected information from all consecutive women diagnosed as invasive breast carcinoma at Seoul National University Hospital and Seoul National University Bundang Hospital from January 2000 to December 2006. Eligibility criteria included complete surgical resection, histologic diagnosis of invasive ductal carcinoma of 1 cm or less (T1a, T1b), or microinvasive carcinoma (T1mic) and no lymph node metastasis (N0) according to the fifth and sixth editions of the American Joint Committee on Cancer (AJCC) Cancer Staging Manual [15]. Both the fifth and sixth editions were used because T staging did not differ between the two staging systems. Patients with a history of previous malignancy of the breast or other sites and who received neoadjuvant chemotherapy were excluded.

### Clinical and biological data acquisition

Data on patient's baseline characteristics and symptoms, mammography results, type of operation and axillary lymph node examinations were collected. The type of operation was divided into mastectomy and breast-conserving surgery, and breast-conserving surgery included lumpectomy, quadrantectomy and excision of the primary tumor. Methods of axillary lymph node examination were classified into sentinel lymph node biopsies and axillary lymph node dissections.

Pathologic information was obtained by reviewing pathology reports on the following variables: tumor size, histologic subtype, modified Bloom-Richardson histologic grade (tubule formation, nuclear pleomorphism, mitotic counts), extent of intraductal carcinoma, lymphatic invasion, venous invasion and tumor border. Immunohistochemical data for standard prognostic biomarkers (ER, PR, HER-2, p53 and Ki-67) were also collected. Immunohistochemical staining was determined from microinvasive foci in cases of T1mic disease. For ER and PR, cases with 10% or more positive staining were grouped as positive. For HER-2, 3+ staining by immunohistochemistry was considered positive. For Ki-67 status, we grouped the cases with 10% or more positive staining as a positive [16]. A 10% cutoff value was also used for p53 expression. Information about treatment undertaken as an adjuvant therapy was also retrieved.

Recurrence free survival (RFS) was calculated as the time from operation to diagnosis of a recurrent disease in the ipsilateral breast, local, regional or distant site. For patients who remained alive and recurrence-free, data were censored at the date of the last follow up. The information of recurrent disease included time of diagnosis, sites of disease (local, regional, systemic), and histologic features of recurrent tumor (ductal carcinoma in situ or invasive ductal carcinoma). Diagnosis of contra-lateral breast cancer was not included in the category of recurrent disease. Overall survival was measured as the time from operation to death.

### Statistical analysis

Pearson's chi-square test was used to assess differences in clinical and biological characteristics between T stage subgroups. The RFS curves were constructed using the Kaplan-Meier method, and the log-rank test was used for a comparison of the survival curves between subgroups according to prognostic factors. We also compared the five-year cumulative survival rate and investigated the association between the probability of death or recurrence and the clinical and biologic features. Multivariate analysis was conducted using Cox's proportional hazard regression model, and our data was shown to satisfy the proportional hazard assumption. A significance level of 0.05 was used for covariate entry.

All statistical analyses were performed using SPSS for Windows software, version 15.0 (SPSS Inc., Chicago, IL). The study was approved by an independent review board at the Seoul National University Bundang Hospital and Seoul National University Hospital.

## Results

### Clinical and histologic characteristics

From January 2000 to December 2006, we identified a total of 3889 women with invasive breast cancer who were diagnosed at the Seoul National University Hospital and Seoul National University Bundang Hospital using the hospital cancer registry. Among these, we excluded 2907 patients (74.7%) with more advanced stage cancer than T1bN0, 76 patients (1.9%) who received neoadjuvant chemotherapy, and 81 patients (2.1%) with recurrent breast cancer. Rest of the patients (n = 450, 11.6%) received diagnosis but did not undergo operation in our hospital therefore were excluded from the study. A total of 375 (9.6%) women were eligible, and 120 patients were classified as T1mic, 93 as T1a, and 162 as T1b. Median age was 49 years (range, 24 to 77 years), and 23 patients (6.1%) were under 35. The majority of the study population (182 patients, 48.5%) was asymptomatic at the time of diagnosis, and these patients were diagnosed by screening mammography. Isolated microcalcification was the most common mammographic finding (149 patients, 40.9%). Pathologic information including hormone receptor and HER2 status was available in 99% of patients. Twenty six percent of patients had HER2 positive tumors, defined by 3+ staining on immunohistochemistry; 67.3% had hormone receptor positive disease; and 15.1% had triple negative disease.

Table 1 shows the baseline characteristics of the three T stage sub-groups. The age distribution between each patient T stage group was similar. In the microinvasive (T1mic) group, there were significantly higher percentages of negative ER and negative PR disease compared to the T1a and T1b groups (57.6% vs. 34.4%, 22.2% for ER, 68.6% vs. 48.4%, 37.7% for PR; p < 0.0001). T1mic tumor also had a higher proportion of HER2 positivity (p < 0.0001); 54 patients (45.8%) in the T1mic group, 21 (22.8%) in T1a, and 21 (13.0%) in T1b. The frequency of positive immunohistochemical staining for p53 was also highest in the T1mic group (p = 0.080 by chi-square, p = 0.037 by Linear-by-Linear association). Ki-67 did not show significant differences between each patient group. Among the 255 patients with T1a or T1b tumor, data of associated DCIS was available in 248 patients. One-hundred and ninety-eight (79.8%) had associated DCIS around the invasive portion, 79 (88.8%) in T1a and 119 (73.6%) in T1b.

**Table 1:** Baseline characteristics of all patients according to T stage

	All patients	T1mic	T1a	T1b	
	(N = 375)	(N = 120)	(N = 93)	(N = 162)	P value
Age at diagnosis					
Median	49	49	47	50	0.297
Range	24 - 77	27 - 74	29 - 77	24 - 73	
Grade^¶^					
1	28(11.2)	.	8(9.0)	20(12.3)	0.126
2	152(60.6)	.	49(55.0)	103(63.6)	
3	71(28.3)	.	32(36.0)	39(24.1)	
ER^a^					
Negative	136(36.5)	68(57.6)	32(34.4)	36(22.2)	<0.001
Positive	237(63.5)	50(42.4)	61(65.6)	126(77.8)	
PR^b^					
Negative	187(50.1)	81(68.6)	45(48.4)	61(37.7)	<0.001
Positive	186(49.9)	37(31.4)	48(51.6)	101(62.3)	
HER2^c^					
Negative	276(74.2)	64(54.2)	71(77.2)	141(87.0)	<0.001
Positive	96(25.8)	54(45.8)	21(22.8)	21(13.0)	
HR/HER2					
HR+HER2-	220(59.1)	42(35.6)	56(60.9)	122(75.3)	<0.001
HR+HER2+	31(8.3)	11(9.3)	9(9.8)	11(6.8)	
HR-HER2+	65(17.5)	43(36.4)	12(13)	10(6.2)	
HR-HER2-	56(15.1)	22(18.6)	15(16.3)	19(11.7)	
p53^d^					
<10%	220(60.6)	60(52.2)	56(63.6)	104(65.0)	0.080
≥10%	143(39.4)	55(47.8)	32(36.4)	56(35.0)	
Ki-67^d^					
<10%	275(75.8)	82(71.3)	72(81.8)	121(75.6)	0.223
≥10%	88(24.2)	33(28.7)	16(18.2)	39(24.4)	

^¶ ^Nuclear/histologic grade was assessed only in T1a and T1b patients.

^a,b^Data of hormone receptor status was available in 373 patients. ;<10%;negative, ≥10%;positive

^c ^HER2 status was determined by immunohistochemistry and was available in 372 patients.; 0,1+,2+;negative, 3+;positive

^d^Data of p53 and Ki-67 was available in 363 patients.

### Loco-regional and adjuvant treatment

178 patients (47.5%) received a mastectomy, and breast-conserving surgery was performed in 197 women (52.5%). For axillary lymph node examinations, more patients (255 patients, 68%) received axillary lymph node dissection than sentinel lymph node biopsies. Among patients who received breast-conserving surgery, 135 patients (68.5%) were treated with adjuvant radiotherapy after surgical resection. The proportion of patients receiving adjuvant whole breast radiotherapy was different in each T stage group: 30 (56.6%) in T1mic, 30 (63.8%) in T1a, and 75 (77.3%) in T1b (p = 0.007).

278 patients (74.1%) received systemic adjuvant therapy. Among patients with hormone-receptor positive disease, 232 (92.4%) received adjuvant endocrine therapy. No differences were observed in the percentage of patients undergoing endocrine therapy between T stage subgroups. Adjuvant chemotherapy was given to 61 (16.3%) patients. Forty-two patients, 34.4% of the patients with ER and PR negative disease, received adjuvant chemotherapy. An anthracycline-based regimen was given to thirty-three patients (54.1% of these 61 patients), and 25 patients were treated with combination chemotherapy of cyclophosphamide, methotrexate, fluorouracil. No patients received adjuvant trastuzumab. T1mic patients were more likely to be candidates for observation without any systemic adjuvant therapy (75.8% in T1mic group, 53.6% in T1a, and 38.7% in T1b, P < 0.0001).

### Recurrence and prognosis

Median follow up duration for all patients was 60.8 months while median RFS duration was not reached as of the time of this writing. The five-year cumulative RFS rate was 97.2%, and recurrent disease was diagnosed in 12 patients during this follow up period. There was no difference in five-year RFS between T1mic, T1a and T1b patients (97.4% for T1mic, 97.8% for T1a, and 96.9% for T1b, p = 0.681) (Table 2). The rate of local or regional recurrence did not show a statistically significant difference between patients who received or not received adjuvant radiotherapy among the patients who received breast conserving surgery (p = 0.871).

**Table 2:** Number of events and five year recurrence free survival according to prognostic factors.

Variable		No. of patients	No. of events	Proportion surviving to 5 years (%)	p value
T stage					
	T1mic	120	4	97.4	0.681
	T1a	93	2	97.8	
	T1b	162	6	96.9	
Age					
	≤ 35	23	3	90.9	0.002
	>35	352	9	97.7	
Grade^¶^					
	1 (low)	28	1	96.3	0.696
	2	152	5	96.6	
	3 (high)	71	2	98.6	
ER^a^					
	negative	136	7	95.1	0.123
	positive	237	5	98.2	
PR^b^					
	negative	187	9	96.0	0.126
	positive	186	3	98.2	
HR					
	ER-PR-	122	7	94.9	0.069
	ER+ and/or PR+	251	5	98.4	
HER2^c^					
	Negative	276	8	97.4	0.772
	Positive	96	3	97.9	
HR/HER2					
	HR+HER2-	220	4	98.6	0.225
	HR+HER2+	31	1	96.8	
	HR-HER2+	65	2	98.5	
	HR-HER2-	56	4	92.6	
HR/HER2					
	HR+ and/or HER2+	316	7	98.4	0.018
	HR-HER2-	56	4	92.6	
p53^d^					
	<10%;	220	4	97.9	0.543
	≥10%	143	6	97.1	
Ki-67^d^					
	<10%	275	6	98.4	0.148
	≥10%	88	4	95.5	

^¶ ^Only T1a, T1b patients were analyzed using nuclear/histolgogic grade.

^a,b^Data of hormone receptor status was available in 373 patients. ;<10%;negative, ≥10%;positive

^c ^HER2 status was determined by immunohistochemistry and was available in 372 patients.; 0,1+,2+;negative, 3+;positive

^d^Data of p53 and i-67 was available in 363 patients.

**Figure 1:**
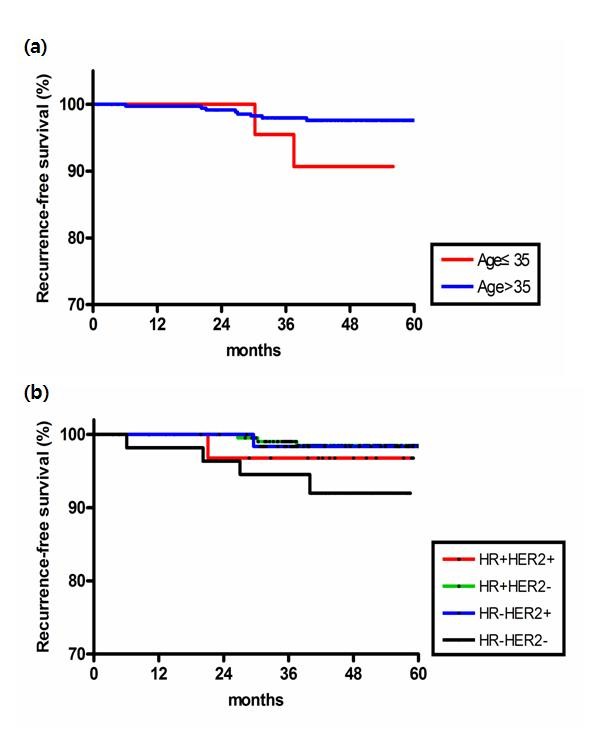
Recurrence-free survival by (a) age (b) breast cancer subtype according to HR and HER2 status

On the univariate analysis, age younger than 35 years and hormone receptor negative disease were risk factors of recurrence. The five-year cumulative RFS rate was 90.9% vs. 97.7% in patients ≤35 and >35 years of age (p = 0.002, Figure 1 A). Each of ER and PR status did not show a significant association with recurrence, but negative status of both ER and PR tend to be associated with shorter RFS (p = 0.069). Patients with both ER and PR negative disease showed a five-year cumulative RFS rate of 94.9%; hormone receptor-positive patients showed a rate of 98.4%. Patients with triple negative disease had a significantly lower RFS rate compared to hormone receptor-positive and/or HER2-positive patients (p = 0.018, Figure 1 B). HER2 status alone, Ki-67 and p53 status were not prognostic factors of RFS. Whether adjuvant chemotherapy was given was not associated with the development of systemic recurrence (p= 0.422).

Cox proportional hazard regression model was constructed using the variables of age, triple negativity, p53 and Ki-67. The independent prognostic factors of recurrence were triple negativity (HR 4.93; 95% CI 1.312-18.519, p = 0.018), and age younger than 35 years (HR 4.91; 95% CI 1.014-23.763, p = 0.048) (Table 3).

**Table 3:** Multivariate analysis: prognostic value of selected factors on recurrence.

	P value	Hazard ratio	95% Confidence interval
Triple negative	0.018	4.93	1.312 - 18.519
Age (≤35)	0.048	4.91	1.014 - 23.763
KI-67	0.481	1.67	0.400 - 6.995
p53	0.819	1.18	0.285 - 4.896

In the analysis of distant disease-free survival, age younger than 35 (p = 0.029) and negative hormone receptor status (p = 0.011) were the convincing poor prognostic factors. Univariate analysis on HER2, Ki67, p53 status and multivariate analysis could not be performed due to the small number of distant metastasis events.

There were twelve patients who experienced recurrence of disease, and their median RFS time was 29.5 months (range 6.1-121.2 months, Table 4). Four patients were diagnosed as T1micN0M0 initially, two patients were T1a, and six were T1b. Three patients were less than 35 years old. Three of 12 patients developed systemic recurrence. All three patients had ER/PR negative disease and developed lung metastases. One patient died during the follow up period. This patient, initially diagnosed with T1bN0M0 breast cancer with ER-PR- and positive HER2, received quadrantectomy with radiation therapy and adjuvant chemotherapy. At 29.5 months after surgical resection, she developed multiple metastases in the lung, skin, and chest wall. She died from breast cancer progression at 40.6 months after surgery.

**Table 4:** Clinicopathologic features of patients with recurrence

No.	Age at diagnosis	T stage	Loco-regional Treatment	Systemic adjuvant treatment	ER	PR	HER2	Site of recurrent disease	RFS (months)
1	31	T1mic	BCS	None	+	+	-	Remnant breast	37.5
2	36	T1mic	BCS, RTx	None	+	-	+	Remnant breast	21.1
3	50	T1mic	BCS, RTx	None	-	-	-	Remnant breast	20.2
4	35	T1mic	Mastectomy	None	-	-	+	Lung	69.9
5	62	T1a	BCS, RTx	None	-	-	Unknown	Remnant breast	31.6
6	29	T1a	Mastectomy	Hormone therapy	+	+	-	Chest wall	30.3
7	52	T1b	BCS	Chemotherapy	-	-	-	Remnant breast	39.9
8	38	T1b	BCS, RTx	Chemotherapy	-	-	+	Lung, Skin	29.5
9	52	T1b	Mastectomy	None	-	-	-	Lung	6.1
10	53	T1b	BCS, RTx	Chemotherapy	-	-	-	Remnant breast	27
11	38	T1b	Mastectomy	Hormone therapy	+	+	-	Remnant breast	26.6
12	60	T1b	Mastectomy	None	+	-	-	Remnant breast	121.2

Abbreviation: BCS, breast conserving surgery; RTx, radiotherapy

## Discussion

The results of this study confirmed an excellent short-term prognosis of lymph-node-negative invasive ductal carcinoma of the breast of 1 cm or less in a Korean population. Among 375 patients, only one patient died of breast cancer and only twelve cases of recurrence were reported. The overall five-year cumulative RFS rate was 97%. Univariate and multivariate analyses revealed that triple negative status and age younger than 35 years were significantly associated with shorter RFS.

There are a number of studies that focus on the prognosis and predictors of recurrence of node-negative early breast cancer. Though many reviews were retrospective and had small populations, high tumor grade was the most consistent factor associated with poor outcome in these studies [6,17-19]. Chia et al. surveyed 430, a relatively large number of patients with T1a,b breast cancer. They noted that patients with grade 3 tumor had shorter 10-year RFS compared to grade 1, 2 patients (with 74% vs. 82%, p = 0.007) [10]. Age, tumor size, lymphovascular invasion and estrogen receptor negativity were also commonly found to be poor prognostic factors [20-23]. In another report, Ki-67 and p53 were considered as significant prognostic factors of survival [24]. More recently, reports have shown that HER2-positive disease is a risk factor of recurrence in node-negative tumors 1cm or smaller [25]. However, most studies on the prognosis of node-negative early breast cancer were performed in Western countries. In contrast, few studies exist regarding the biology and prognosis of Asian breast cancer patients.

In the present study, triple negative status and age younger than 35 were the only independent prognostic factors of recurrence. Other studies have reported similar results in which younger patients showed poorer prognosis than older patients. These authors suggested that poor prognosis observed in young patients under the age of 35 was related to adverse characteristics of their disease, such as a high proportion of node-positive disease, a high proportion of negative estrogen hormone receptor, and a high proliferation index. However, age younger than 35 years remained an independent poor prognostic factor in the multivariate analysis. However, an analysis of the prognosis of a specific subgroup with tumor sizes of 1 cm or less was not performed in this study [9]. In our patients, there were no differences in pathologic characteristics of hormone receptor status or HER2 status according to age group (Table 5). Our data suggest that age younger than 35 is an independent risk factor for recurrence, even in patients with node-negative breast cancer 1 cm or less in size. Therefore, young age less than 35 should be regarded as a poor prognostic factor when adjuvant therapy is considered. This is important, especially for Asian patients, because the proportion of young women in Asian countries is higher than in Western countries [5,26].

**Table 5:** Pathologic characteristics according to age group

	Age ≤ 35 (%)	Age > 35 (%)	P value
Hormone receptor (HR)			
ER+ and/or PR+	14 (60.9)	237 (67.7)	0.498
ER-PR-	9 (39.1)	113 (32.3)	
HER2^a^			
Negative	8 (34.8)	88 (25.2)	0.328
Positive	15 (65.2)	261 (74.8)	
HR/HER2			
HR+ and/or HER2+	19 (82.6)	297 (85.1)	0.763
HR-HER2-	4 (17.4)	52 (14.9)	
p53			
<10%;	13 (59.1)	207 (60.7)	1.000
≥10%	9 (40.9)	134 (39.3)	
Ki-67			
<10%	15 (68.2)	260 (76.2)	0.441
≥10%	7 (31.8)	81 (23.8)	

^a^HER2 status was determined by immunohistochemistry and was available in 372 patients.; 0,1+,2+;negative, 3+;positive

The grade of HER2 overexpression which was a poor prognostic factor in other studies [24,25,27,28] did not have an effect on RFS in our study. This discrepancy can be attributed to the fact that HER2 fluorescence in situ hybridization (FISH) was not performed in most of the patients. There is a possibility that HER2 FISH positive disease was classified as HER2 negative based on immunohistochemical staining only. A literature show that quite a few patients with moderate expression (+2) of HER2 in immunohistochemistry had HER2 gene amplification at FISH [29]. Another explanation for the lack of an association between HER2 status and recurrence may be the inclusion of T1mic disease in the analysis. Patients with T1mic disease have a higher proportion of HER2 positive disease but a very low potential of systemic metastasis or recurrence. In our series, endocrine unresponsiveness and positive HER2 immunohistochemical staining (+3) were more common in T1mic tumor than T1a or T1b disease. The reason for high HER2 positive rate in T1mic tumor is not clearly understood, but other literatures also report the similar findings [12,30].

More than half of all patients received breast-conserving surgery in our study, but 31.5% of these patients, mainly T1mic or T1a, did not receive post-operative radiotherapy because the tumor size was extremely small. The local recurrence rate of this group was 3.2% (2 in 62 patients), which was not high compared to the patients who received adjuvant radiotherapy (2.9%, 4 in 135 patients). This may have resulted from the very low incidence of recurrence itself; hence, a longer duration of follow up and a larger number of patients are necessary before definitive conclusions can be made regarding the role of post-operative radiotherapy in very small breast cancer.

Our study has several limitations. First, recurrence of early breast cancer is known to be discovered not only during the first decade of follow up, but also during the second decade or even later. Therefore, our duration of follow up was not sufficient for an evaluation of breast cancer prognosis regarding the overall survival and long-term recurrence rate, especially in endocrine-responsive disease. However, an analysis of the short-term recurrence rate and risk factors would be valuable for the identification of specific patient groups who require systemic adjuvant chemotherapy after surgery. Second, other important pathological information, such as mitotic activity [31,32] and vascular invasion [33] were not included in this analysis. Third, many patients received systemic adjuvant therapy according to their hormone receptor status and other clinical factors. Therefore, we cannot conclude whether the pattern of recurrence reflects the natural history of specific subgroups or a response to the adjuvant therapies given. In fact, 92.4% of hormone receptor positive patients received endocrine therapy while 34.5% of hormone receptor negative patients were treated with adjuvant chemotherapy. It is within the realm of possibility that good prognosis of a patients group with positive ER or PR status was caused by the efficacy of the adjuvant hormonal therapy. In addition, ER and PR positivity was defined as 10% or more staining unlike definitions used in recent studies focusing on breast cancer subtypes, which classified tumors as hormone-receptor positive if 1% or more of tumor nuclei were stained [34]. However, we regarded the tumor with 10% or more positive staining as hormone receptor-positive disease, because the pathology report of our patients, especially who were diagnosed in the early 2000s, used the previous classification standard. There may be a possibility that tumors with positive staining of ER and or PR between 1-9% may have been classified as HR negative.

Finally, a definite conclusion pertaining to the subgroup of patients who required systemic adjuvant treatment could not be determined because a multivariate analysis could not be performed for predictors of systemic recurrence due to the low number of events.

## Conclusion

We confirmed that patients with a tumor size of 1cm or less of node-negative invasive ductal carcinoma of the breast have excellent short term prognosis. Studies with a larger sample size and a longer follow up duration are needed for confirmation of this result, but patients younger than 35 years of age and triple negative disease were identified to be the highest risk group of recurrence.

## Competing interests

The authors declare that they have no competing interests.

## Authors' contributions

JH Kwon, JH Kim conceived of the study, and participated in its design of the study, coordination, and performed data acquisition, statistical analysis, and interpretation. SYP, EKC, SWK, IAK, TYK, IAP, DYN, YJB and SWH have been involved in revising it critically for important intellectual content. JH Kwon, YJK, KWL, DYO, JH Kim and SAI participated in drafting the manuscript. SAI, TYK, YJB have given final approval of the version to be published. All authors read and approved the final manuscript.

## Pre-publication history

The pre-publication history for this paper can be accessed here:

http://www.biomedcentral.com/1471-2407/10/557/prepub
